# Prevalence and Associated Factors of Intestinal Parasitic Infections among Food Handlers at Prison, East and West Gojjam, Ethiopia

**DOI:** 10.1155/2019/2101089

**Published:** 2019-01-06

**Authors:** Azmeraw Asires, Moges Wubie, Alemayehu Reta

**Affiliations:** ^1^Jimma University, Department of Animal Science, Jimma, Ethiopia; ^2^Debre Markos University, College of Health Sciences, Department of Public Health, Debre Markos, Ethiopia; ^3^Debre Markos University, College of Health Sciences, Department of Medical Laboratory Science, Debre Markos, Ethiopia

## Abstract

**Introduction:**

One of the top ten major public health problems in developing countries including Ethiopia is the intestinal parasitic infection. Most of the time, intestinal parasitic infections do not show clinical signs and symptoms and also have a number of potential carriers, such as food handlers, which makes it too difficult to eradicate and control.

**Objective:**

The aim of this study is to assess the prevalence and associated factors of intestinal parasitic infection among food handlers at prison, East and West Gojjam, Ethiopia, 2017.

**Methods:**

An institution-based cross-sectional study design was conducted at East and West Gojjam prison. A total of 416 study participants, with a response rate of 82.7%, were included in the study for both stool exam and questioner. Data were collected using a structured questionnaire, and the sample was collected and examined based on the standard parasitological procedure. Epi data Version 3.1 was used to enter data, and SPSS version 20 was used to analyze the data.

**Results:**

The overall prevalence of intestinal parasitic infections in the present study was 61.9%. The most prevalent parasite was *A. lumbricoides* (157 (45.6%)). Protozoan infection was higher than helminth infection. Multiple intestinal infections were identified; among study participants, 34.6% had double infection. The most significant associated factors of intestinal parasitic infections were fingernail status, residence, information about food contamination related to intestinal parasitic infection, income, and handwashing before having contact with food and after toilet with water only.

**Conclusions:**

A high proportion of intestinal parasitic infection was detected among food handlers working at East and West Gojjam prison. Training must be given to the food handlers on personal hygienic conditions (finger trimming, handwashing after toilet and before having contact with food with water and soap, etc.).

## 1. Background

Parasitic diseases are widely distributed throughout the world, and they continue to be problematic in developed, developing, and less developed countries. The disease-causing parasites may produce serious infections and occasionally the death of their hosts [1].

Most of the time, intestinal parasitic infections (IPIs) do not show clinical signs and symptoms and also have a number of potential carriers, such as food handlers, which makes it too difficult to eradicate and control [2]. The spread of disease by food handlers is a common and persistent problem worldwide [3]. Those food handlers with poor personal hygiene working in the food service settings can easily be infected with enteric pathogens [4], possibly causing fecal contamination of foods with their hands during food preparation, and finally may be implicated in the transmission of many infections to the public in the local community [5].

Studies conducted around the world such as those in Pakistan, Kenya, Iran, Saudi Arabia, and Nigeria revealed that there is an increased prevalence of IPIs caused by *Entamoeba histolytica/dispar*, *Ascaris lumbricoides*, *Giardia lamblia*, and hookworm [6–10].

Several studies about the prevalence of intestinal parasites were conducted, which showed protozoan infections were more common than helminth infections [11–14]. The reason for this is that the transfer of protozoans is much easier than the transfer of the eggs or larvae of worms [15, 16] and the use of self-medication is restricted to helminths [17].

The overall prevalence of intestinal parasitic infection in the study conducted in Teda Health Center, Northwest Ethiopia, was 62.3%. *A. lumbricoides* was the most predominant parasite (23.2%) followed by *Giardia intestinalis* (12.4%), *Entamoeba histolytica/dispar* (4.6%), *Schistosoma mansoni* (8.9%), hookworm (6.6%), *Hymenolepis nana* (1.5%), *Enterobius vermicularis* (0.4%), and *Strongyloides stercoralis* (0.2%) [18].

**Figure 1:**
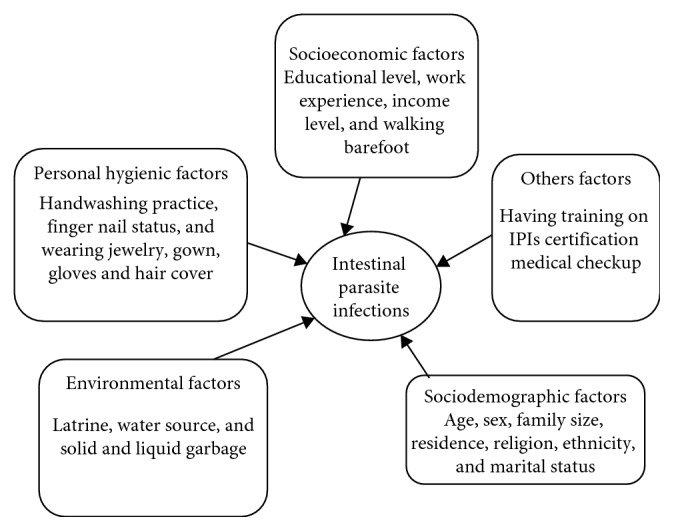
Conceptual framework of food handlers who are working at East and West Gojjam prison, Ethiopia, 2017.

Concerning the risk factors, the study conducted in different parts of Ethiopia stated that there is a significant association between poor handwashing practice after toilet with soap and water and sociodemographic variables (sex, age, educational status, and years of service) and IPI [3, 17, 18, 19]. For this study, we prepare a conceptual framework for the risk factors (Figure 1).

Even though numerous studies have been conducted on the prevalence of intestinal parasites in Ethiopia, there are still a number of localities in the country including the present study area, East and West Gojjam prison, for which information about the prevalence and associated factors of intestinal parasitic infections is not available. Therefore, the purpose of this study was to obtain information about the prevalence and associated risk factors from food handlers at East and West Gojjam prison, Ethiopia.

## 2. Methods

**Figure 2:**
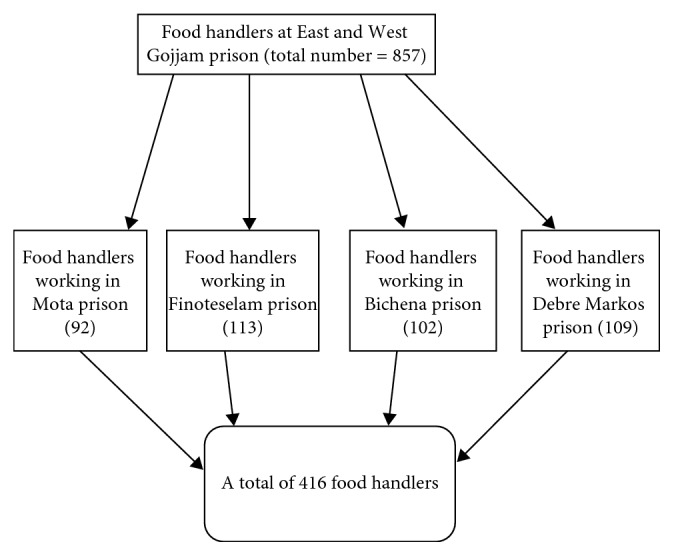
Sampling procedure of food handlers who are working at East and West Gojjam prison, Ethiopia, 2017.

An institution-based cross-sectional study was conducted at the prison of East and West Gojjam to assess the prevalence and associated factors of intestinal parasitic infection among food handlers at the prison from March 12–April 09, 2017. Sample size of each prison was determined proportionally. A simple random sampling technique was used to draw samples from each prison. The list of food handlers from each prison was taken from the respective prison administrative. A total of 416 food handlers participated. Food handlers who were taking antihelminthics and antiprotozoans during data collection were excluded. Then, samples were taken and used for both questionnaires and stool examination (Figure 2).

## 3. Data Collection Instrument

### 3.1. Questionnaire

A pretested and structured questionnaire was used to collect data. Data were collected by interviewing and observing the participant. The questionnaire was coded and prepared in English and then translated into Amharic and back to English to see its consistency with PI. Factors associated with IPI such as sociodemographic factor, socioeconomic factor, personal factors, and medical checkup were included in the questionnaire.

### 3.2. Observation

The status of the fingernails and information on wearing hair cover/cap, glove, and gown and whether the participants were on barefoot or not were observed.

### 3.3. Personnel

A total of 15 data collectors were employed. Seven data collectors/interviewers (who completes grade 10 and above), 4 supervisors (diploma and above), and 4 laboratory technicians (BSc) were employed. At each prison site, one supervisor and one BSc graduate were present in the laboratories for stool examination, and two data collectors each at Finoteselam, Debre Markos, and Bichena and one data collector at Mota prison center were assigned.

## 4. Parasitological Procedures

A direct wet mount with normal saline (0.85% NaCl solution) was prepared at the study site and observed for the presence of motile intestinal parasites, trophozoites, and eggs under a light microscope at 10x and 40x magnification. Lugol's iodine staining was also used to observe the cysts of intestinal parasites. The concentration technique was used to detect the eggs of intestinal parasites that are excreted only intermittently or in small numbers, like *Taenia* or *Schistosoma* species [20].

## 5. Quality Control of Data

Training was given for two days to the data collectors and supervisors before data collection. Before data collection, 10% of pretest was done at the prison. The quality of collected data was checked by the principal investigator and/or supervisors daily. Before analysis, cross-checking was done on the data by principal investigators to ensure the quality of the data.

## 6. Data Analysis

Data were coded, entered into Epi Data version 3.1, and exported to SPSS version 20 statistical package for analysis. Frequency, percentages, tables, and figures were used to describe the findings. Mean and the standard deviation were used to summarize the data. The association between dependent variables and independent variables was determined by using odds ratio with 95% confidence interval and at 0.05 *p* values. Variables which have a *p* value less than 0.2 were taken for multivariate analysis. A *p* value less than 0.05 was considered as significant.

## 7. Result

From 416 study participants, the participants with a response rate of 82.7% were included in the study. The median age of the study subjects was 28.00 years (+SD), SD = 10.590, the age ranged from 14 to 60 years, and 70% (241) of the study participants were male.

## 8. Personal Hygiene and Environmental Characteristics of Study Participants

In handwashing practices, 272 (79.1%) food handlers had a habit of handwashing after toilet, while 226 (83.1%) food handlers had the habit of handwashing with soap and water after toilet. Almost all food handlers (342 (99.4%)) had a habit of handwashing before having contact with food items. Majority of the study participants (food handlers) (222 (64.5%)) have had no medical checkup previously, including stool examination. All food handlers working at the prison center were not certified. 148 (43.0%) food handlers trimmed their fingernails well, 112 (32.6%) trimmed their fingernails partially, and the rest (84 (24.4%)) did not trim their fingernails (Table 1). Sanitary facility of study participants working in the entire four prison centers had a latrine at the workplace but none of them had handwashing facility inside the latrine. Majority of participants (282 (81.9%)) had pipe water access, and 160 (46.5%) used burning solid disposal (Table 2).

## 9. Prevalence of Intestinal Parasitic Infections

**Figure 3:**
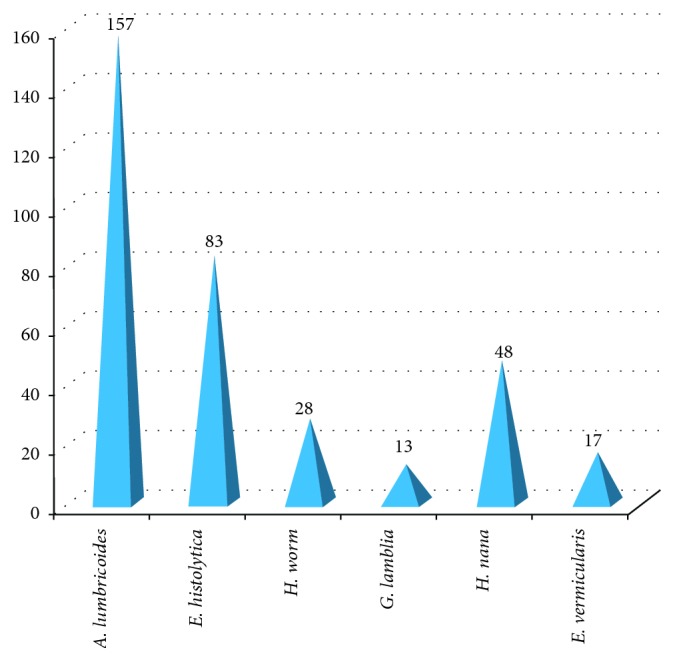
Prevalence of intestinal parasitic infections at East and West Gojjam prison from March 01–April 09, 2017.

**Figure 4:**
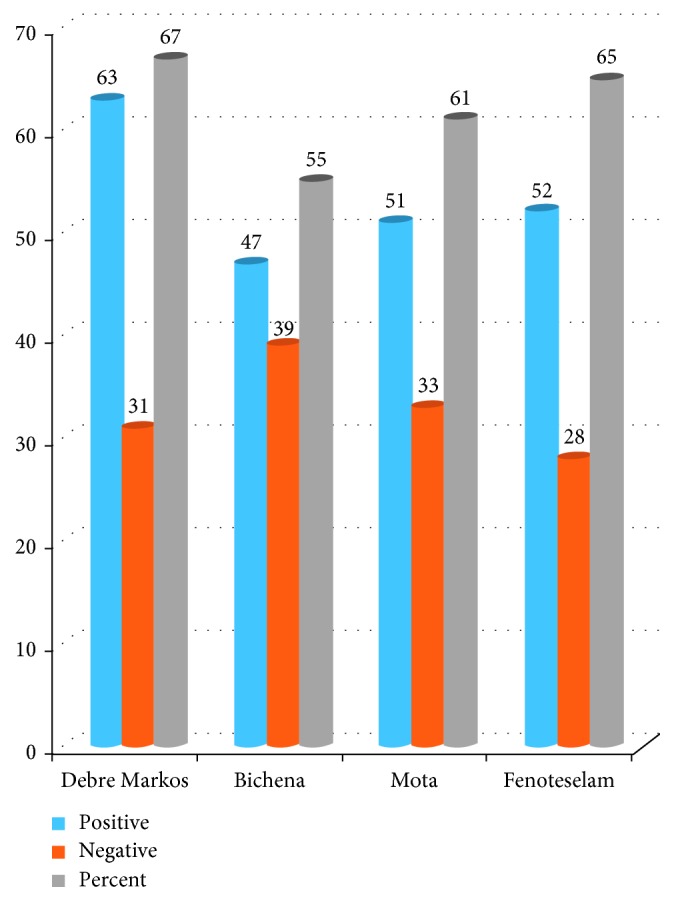
Prevalence of intestinal parasite in each prison centers at East and West Gojjam prison from March 01–April 09, 2017. NB: “percent” indicated in the legend of the chart shows positivity.

The prevalence of intestinal parasite infections among food handlers who were working at East and West Gojjam prison is summarized in Table 4. The overall prevalence of intestinal parasites to at least a single infection was 213 (61.9%). The most prevalent parasites were *A. lumbricoides* (157 (45.6%)), followed by *E. histolytica* 83 ((24.1%)), *H. nana* (48 (14.0%)), hookworm (28 (8.1%)), *E. vermicularis* (17 (4.9%)), and *G. lamblia* (13 (3.8%)) (Figure 3). The highest prevalence of intestinal infection was recorded at Debre Markos prison (63 (67%)), followed by that at Fenoteselam (52 (65%)) and Mota (51 (61%)). Out of four prisons located at East and West Gojjam, the lowest prevalence of IPIs was found at Bichena prison (47 (55%)) (Figure 4).

## 10. The Level of Infections

**Figure 5:**
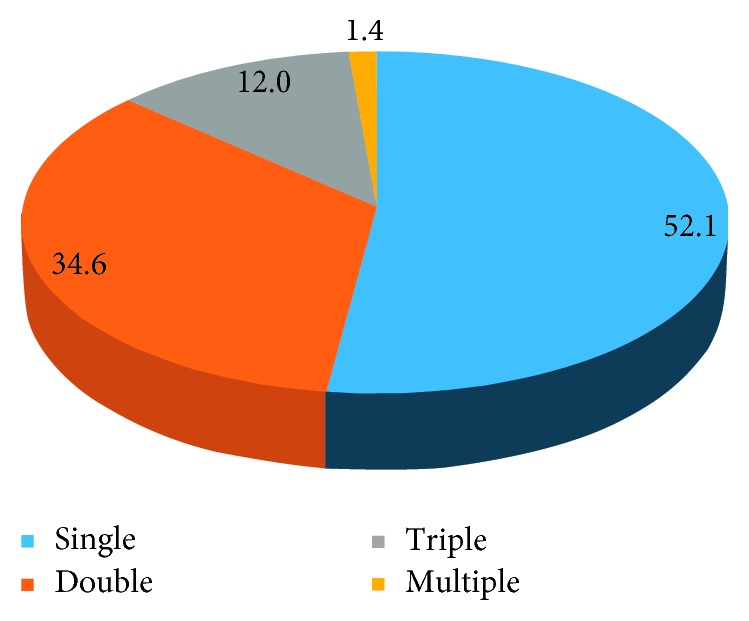
Level of intestinal parasitic infections among study participants at East and West Gojjam prison from March 01–April 09, 2017.

Out of total participants, 75 (34.6%) had double infection, 26 (7.3%) had triple infection, and 3 (0.8%) had multiple infection. Multiple infection comprises four intestinal parasite specious levels of infestation (Figure 5).

## 11. Binary and Multiple Logistic Regression Results of Study Participants

Binary logistic regression analysis was performed to assess the independent effect of each factor. Then, multivariate logistic regression was performed for factors which are found to be associated with binary logistic regression analysis. The study participants' residence, monthly income, habit of handwashing after toilet with water, information about food contamination related to IPIs, habit of handwashing with water alone before having contact with food , and fingernail status were found to be the independent factors of IPIs at a significance level of 0.05. The residence was found to be a strong significant predictor of IPI; rural residents are more likely to be infested with intestinal parasite than the urban residents (AOR = 4.675, 95% CI = 1.533–14.255, *p*=0.007). Monthly income was also another strong determinant factor for IPI; when compared with food handlers who earned greater than 2000 ETH Birr, those individuals with monthly income ranging from 500 to 999 ETH Birr are more likely to be infested with IP (AOR = 45.891, 95% CI = 4.070–517.485, *p*=0.002). Handwashing with water only after toilet was a significant risk factor for IPI. Those food handlers who washed their hands with water only were more likely infested than those food handlers who washed their hands with water and soap after toilet (AOR = 6.734, 95% CI = 1.291–35.120, *p*=0.024). Similarly, handwashing with water alone before having contact with food was a significant risk factor of IPI. Food handlers who washed their hand with water only before having contact with food were more likely to be infested with IP than those who washed their hands with water and soap before having contact with food (AOR = 7.334, CI = 1.815–29.637, *p*=0.005) (Table 3). Having information about food contamination, especially intestinal parasite, was strongly associated with IPIs. Those food handlers who had no information had greater risk for IPIs (AOR = 11.947, CI = 3.681–38.77, *p* ≤ 0.001). Fingernail status was the other strongly associated factor for IPI. When compared with trimmed fingernail status, those who trimmed fingernail partially had greater risk of IPIs (AOR = 9.959; CI = 3.182–31.173, *p* ≤ 0.001) (Table 4).

## 12. Discussion

The overall prevalence of intestinal parasite in the present study was 213(61.9%). This finding is comparable with the study conducted in Teda Health Center, Northwest Ethiopia (62.3%) [18]. As compared with the research done at Hamadan, this finding was lower than that of the study conducted in Hamadan, Western Iran (74%) [8]. However, this finding was higher than that of a study conducted at among food handlers at University student cafeteria Addis Ababa, Ethiopia (45.3%) [21], in Yebu town, Southwest Ethiopia (44.1%) [22], and in Southern Brazil (28%) [23]. Possibly, the difference might be due to the geographical difference, the living, and the socioeconomic status of the study participants.

The leading/predominant intestinal parasite in the present study was *A. lumbricoides* (45.6%). This is comparable with that of the study conducted in Teda Health Center, Northwest Ethiopia [18], and Yebu town, Southwest Ethiopia [22].

The second most prevalent intestinal parasite in this study was *E. histolytica* (24.1%) which is lower than that of a research conducted at Addis Ababa university student cafeteria (70.8%) [21], in Pakistan (48.86%) [6], and in Nigeria (52.4%) [10]. The possible difference might be due to the geographical difference, the living, and the socioeconomic status. But, this finding was also higher than that of a research conducted at Teda Health Center, Northwest Ethiopia (4.6%) [18], in Kenya (12.5%) [7], in Hamadan, Western Iran (14.5%) [8], and in Saudi Arabia (2.78%) [9]. The discrepancy might be due to the living standard and geographical difference.

In the present study, the prevalence of protozoan intestinal parasitic infestation (27.9%) was lower than helminths intestinal parasitic infection (72.6%). This result disagrees with the results of the studies conducted in the Omdurman area of Sudan [13], South of Tehran, Iran [11], and Islamic Republic of Iran [14].

The prevalence of double intestinal parasitic infections was 34.6%, which was much higher than that reported from Northeast Thailand (5.9%) [24].

Residences of food handlers were significantly associated with IPIs. Food handlers who were rural residents were more likely to be exposed to parasitic infections than food handlers who were urban residents; a possible reason for this might be due to the living condition related to sanitation and economic difference.

Those food handlers who do not trim their fingernails completely/who trimmed partially were significantly associated with parasitic infection than those food handlers who trimmed their fingernails completely. This finding was in agreement with a study conducted at Arba Minch student Cafeteria [19] and Yebu town Southwest Ethiopia [22].

Another significantly associated factor with parasitic infection was those having information about food contamination related to intestinal parasite infection had positive association than those who had no information. The possible explanation might be the level of awareness about food contamination related to IPI in food handlers who had no information was lower than who had.

Food handlers who were working at East and West Gojjam prison and earned 500–999 ETH Birr were more likely to be exposed to intestinal parasitic infection than those who earned >2000 ETH Birr.

Those food handlers who washed hands with water only before having contact with food were significantly associated with IPIs than those who washed their hands with water and soap.

Those food handlers who washed their hands after toilet with water only were more likely exposed to intestinal parasitic infection than those who washed their hands with water and soap. This finding was in agreement with the study conducted at Gondar University [17] and at Arba Minch university student cafeteria [19].

Other risk factors like wearing shoe and gown, medical checkup, water source, and sex were not significantly associated with parasitic infection.

However, this study has delivered valuable information concerning the prevalence and associated factors of intestinal parasitic infection among food handlers at prison, and there were some limitations that could be addressed in the research. One of the limitations of this study was the parasitological techniques used to diagnose different parasites were less sensitive. This can directly affect the prevalence of parasites. The reason for such compromisation was due to a limited budget allowed to perform the research.

## 13. Conclusions and Recommendations

A high prevalence of intestinal parasitic infection was observed among food handlers working at East and West Gojjam prison. In this study, intestinal parasites such as *A. lumbercoides*, *E. histolytica*, hookworm, *H. nana*, *E. vermicularis*, and *G. lamblia* were identified. This study investigated that double and multiple infections were higher than those of the studies conducted before. All prisons had no handwashing facilities inside the latrine, and all food handlers never use hand gloves.

Factors such as fingernail status, monthly income, handwashing with water only before having contact with food and after toilet, and having information about food contamination related to intestinal parasitic infection were significantly associated with the occurrence of intestinal parasitic infection in our study area. Based on the results of the study, the following recommendations are suggested: Increasing the knowledge and awareness of food handlers via providing information about food contamination related to intestinal parasitic infections. Training must be given to food handlers on personal hygienic conditions (like finger trimming and handwashing after toilet and before having contact with food with water and soap). The prison shall construct handwashing facilities inside the latrine, and it is better if the prison employees were trained and certified food handlers. Further studies should be undertaken on the prevalence of intestinal parasite infections and associated risk factors. Continuous checkup of food handlers should be mandatory to alleviate the problem by the concerned body.

## Figures and Tables

**Table 1:** The hygiene practice of food handlers working at East and West Gojjam prison from March 12–April 09, 2017.

Characteristics	No	%
*Handwashing after toilet*		
Yes	272	79.1
No	72	20.9
** **Using water only	46	16.9
** **Using soap	226	83.1
*Handwashing before having contact with food*		
Yes	342	99.4
No	2	0 .6
** **Using water	133	38.9
** **Using soap	209	61.1
*Medical checkup*		
Yes	122	35.5
No	222	64.5
*Fingernail status*		
Trimmed	148	43.0
Semitrimmed	112	32.6
Not trimmed	84	24.4
*Wearing jewelry*		
Yes	71	20.6
No	273	79.4
*Wearing gown*		
Yes	119	34.6
No	225	65.4
*Wearing head cap*		
Yes	25	7.3
No	319	92.7

**Table 2:** Environmental factors for food handlers working at East and West Gojjam prison from March 1–April 09, 2017.

Characteristics		No	%
Water source	Pipe water	282	81.9
	Well water	37	10.8
	River	25	7.3

Solid-liquid garbage	Burning	160	46.5
	Pit	139	40.4
	Open field	45	13.1

**Table 3:** Relationships between sociodemographic characteristics and intestinal parasitic infections among food handlers working at East and West Gojjam prison from March 01–April 09, 2017.

Characteristics	Positive (no. (%))	Negative (no. (%))	Crude OR ((95% CI))	Adjusted OR (95% CI)	*p* value
Residence					
** **Rural	110 (88)	15 (12)	8.259 (4.527–15.062)	4.675 (1.533–14.255)	0.007
** **Urban	103 (47)	116 (53)	1		

Monthly income level					
** **<500	117 (64)	66 (36)	0.834 (0.519–1.41)	0.755 (0.265–2.156)	0.012
** **500–999	91 (68)	42 (32)	12.515 (2.759–56.761)	45.891 (4.070–517.4)	0.600
** **1000–2000	2 (13)	14 (87)	5.364 (1.403–20.503)	0.493 (0.034–7.200	0.001
** **>2000	3 (25)	9 (75)	1		0.605

Information about IPI					
** **Yes	28 (20)	112 (80)	11.282 (6.329–20.109)	11.947 (3.681–38.77)	<0.001
** **No	110 (85)	19 (15)	1		

**Table 4:** Relationship between hygiene practice of food handlers who are working at East and West Gojjam prison and intestinal parasitic infections from March 01–April 09, 2017.

Characteristics	Positive (no. (%))	Negative (no. (%))	Crude OR (95% CI)	Adjusted OR (95% CI)	*p* value
Handwashing after toilet					
Yes	143 (53)	129 (47)	0.032 (0.008–0.132)	6.548 (1.263–33.962)	0.025
No	70 (97)	2 (3)	1		
** **Using water only	38 (83)	8 (17)	5.474 (2.445–12.255)	6.734 (1.291–35.120)	0.024
** **Using soap	105 (46)	121 (54)	1		

Handwashing before having contact with food					
** **Using water	98 (74)	35 (26)	2.333 (1.455–3.742)	7.334 (1.815–29.637)	0.005
** **Using soap	114 (55)	95 (45)	1		

Fingernail status					
Trimmed	50 (34)	99 (66)	1		
Semitrimmed	92 (83)	19 (17)	11.035 (5.572–21.85)	9.959 (3.182–31.173)	<0.001
Not trimmed	71 (84)	13 (16)	1.116 (0.517–2.410)	0.694 (0.193–2.499)	0.544

## Data Availability

The research data used to support the findings of this study are included within the article.

## List of Abbreviations

AOR: Adjusted odds ratio
BSc: Bachelor of science
CI: Confidence interval
ETH: Ethiopian
IPI: Intestinal parasitic infection
NaCl: Sodium chloride
P: Probability
PI: Principal investigator
SD: Standard deviation
SPSS: Statistical Package for Social Science.
